# Evidence of biomechanical and collagen heterogeneity in uterine fibroids

**DOI:** 10.1371/journal.pone.0215646

**Published:** 2019-04-29

**Authors:** Friederike L. Jayes, Betty Liu, Liping Feng, Nydea Aviles-Espinoza, Sergey Leikin, Phyllis C. Leppert

**Affiliations:** 1 Department of Obstetrics and Gynecology, Duke University School of Medicine, Durham, North Carolina, United States of America; 2 Department of Pathology, Duke University School of Medicine, Durham, North Carolina, United States of America; 3 Department of Orthopedics, Duke University School of Medicine and Duke University School of Engineering, Durham, North Carolina, United States of America; 4 Bone and Matrix Biology in Development and Disease, Division of Intramural Research, Eunice Kennedy Shriver National Institute of Child Health and Human Development, Bethesda, Maryland, United States of America; University of Crete, GREECE

## Abstract

**Objective:**

Uterine fibroids (leiomyomas) are common benign tumors of the myometrium but their molecular pathobiology remains elusive. These stiff and often large tumors contain abundant extracellular matrix (ECM), including large amounts of collagen, and can lead to significant morbidities. After observing structural multiformities of uterine fibroids, we aimed to explore this heterogeneity by focusing on collagen and tissue stiffness.

**Methods:**

For 19 fibroids, ranging in size from 3 to 11 centimeters, from eight women we documented gross appearance and evaluated collagen content by Masson trichrome staining. Collagen types were determined in additional samples by serial extraction and gel electrophoresis. Biomechanical stiffness was evaluated by rheometry.

**Results:**

Fibroid slices displayed different gross morphology and some fibroids had characteristics of two or more patterns: classical whorled (n = 8); nodular (n = 9); interweaving trabecular (n = 9); other (n = 1). All examined fibroids contained at least 37% collagen. Tested samples included type I, III, and V collagen of different proportions. Fibroid stiffness was not correlated with the overall collagen content (correlation coefficient 0.22). Neither stiffness nor collagen content was correlated with fibroid size. Stiffness among fibroids ranged from 3028 to 14180 Pa (CV 36.7%; p<0.001, one-way ANOVA). Stiffness within individual fibroids was also not uniform and variability ranged from CV 1.6 to 42.9%.

**Conclusions:**

The observed heterogeneity in structure, collagen content, and stiffness highlights that fibroid regions differ in architectural status. These differences might be associated with variations in local pressure, biomechanical signaling, and altered growth. We conclude the design of all fibroid studies should account for such heterogeneity because samples from different regions have different characteristics. Our understanding of fibroid pathophysiology will greatly increase through the investigation of the complexity of the chemical and biochemical signaling in fibroid development, the correlation of collagen content and mechanical properties in uterine fibroids, and the mechanical forces involved in fibroid development as affected by the various components of the ECM.

## Introduction

Uterine fibroids, also called leiomyomas, are benign tumors that arise from myometrium. Seventy to eighty percent of women will develop uterine fibroids by age 50 [1], and many suffer from pressure, pain, infertility, and severe bleeding. While these widespread tumors have been the subject of basic and translational studies for decades [2–5], their molecular pathobiology remains elusive and as a result current treatment options are limited.

These tumors are fibrotic and enveloped by a pseudocapsule that separates the benign tumor tissue from the surrounding myometrium. It has been shown by different techniques that uterine fibroids are two to four-fold stiffer than myometrium [6–9]. The stiffness of fibroids results from their abundant extracellular matrix (ECM) which includes large amounts of glycosaminoglycans and more importantly large amounts of disordered, highly cross-linked interstitial collagens [7, 8, 10–15]. In addition, studies have linked the increased stiffness to altered biomechanical signaling in the tumors. [7, 8].

Heterogeneity of uterine fibroids is often not appreciated and therefore not considered in the design and conduct of basic, translational, and clinical studies. As a result, there are numerous shortcomings in understanding the pathobiology of these tumors. Without clear characterization of samples, it is challenging to define and compare phenotypes. An appreciation of sample differences will better enable comparisons between studies and improve understanding of these benign but problematic fibrotic tumors. Heterogeneity has been documented on the genetic/genomic, proteomic, metabolomic and histologic level. [2, 16–26]. During our ongoing research on the development of treatments for uterine fibroids, we have noted additional heterogeneity. The gross pathologic appearance of uterine fibroids is usually described as well-circumscribed, firm, white to greyish whorled tissue [27]. However, we have observed a wide range of gross appearances and variability in fibroid stiffness. Here we characterize the intra- and inter-fibroid variations we observed by gross appearance, mechanical properties, and content of interstitial cross-linked helical collagens which provide stiffness to fibroids.

## Methods

### Collection of fibroid tissues for appearance, amount of fibrosis and stiffness

Our studies were approved by the Duke Institutional Review Board. Women over 18 years of age with a diagnosis of uterine fibroids provided written consent. Fibroid tissue from 20 tumors was obtained post-hysterectomy in nine subjects. All tumors were considered to be common benign uterine fibroids by the examining pathologist and none of the tumors were from patients with the hereditary leiomyomatosis and renal cell cancer (HLRCC) syndrome. The fibroids varied in size from three to eleven centimeters in diameter. Tissue from one subject was excluded from the analysis because the tissue was recalled by the pathologist for further examination. Therefore, 19 fibroids from eight subjects were included in the analysis. Immediately following surgery, we obtained slices (cross sections) of approximately 1 cm thickness from each fibroid. The tissues were transported to the laboratory and washed as described previously [6]. The gross appearance of the cut surface was observed and recorded; photographs were successfully obtained for 18 fibroids. Tissue slices were then cut into smaller pieces and either snap frozen at -80°C for mechanical stiffness studies or fixed in formalin for histology.

### Masson trichrome staining

Fixed tissues were paraffin embedded, sectioned (5μm), and stained with Masson trichrome in the Duke Histology Core Laboratory. Masson trichrome is commonly used to differentiate collagen (stained blue-green) from surrounding muscle cells (stained red). Briefly, slides were stained with Weigert’s iron hematoxylin followed by Ponceau acid fuchsin. After treatment with phosphomolybdic-phosphotungstic acid slides were stained with Light Green in acetic acid. Whole slides were scanned at 20x (Aperio Scanscope, Leica Biosystems Inc., Buffalo Grove, IL). Aperio ImageScope and Adobe Photoshop (Adobe Systems Inc., San Jose, CA) CS6 software was used to analyze the entire section on each slide. The quantity of blue-green pixels as a proportion of total pixels was used to determine percent (%) collagen as previously described [6, 9].

### Mechanical stiffness studies

Each fibroid described above was evaluated for the biomechanical property of stiffness by rheometry as described previously [6]. Briefly, from each fibroid, two to three random 5 mm diameter punches were obtained (n = 44) and measured dynamically to determine sample stiffness (complex shear modulus Pascal [Pa] at 10 rad/sec) taking into account both the viscous and elastic behavior of the tissue. Freezing and thawing and repeat measures of fibroid tissue did not affect stiffness measurements [6]. The punches from each fibroid were used to calculate variability within fibroids. Samples from each fibroid were averaged to calculate fibroid stiffness for comparison among fibroids. Five subjects had more than one fibroid (2–4 fibroids per subject) and average stiffness per subject was calculated for comparison among subjects.

### Determination of type I, III, and V collagen content

#### Uterine fibroid samples

From five additional consented subjects, we obtained fibroid tissue samples immediately following hysterectomy as described previously [28]. Fibroid size ranged from 4 to 12.5 cm and tissue samples (1 cm^3^) were obtained within 1 cm from the fibroid edge (E) and from the center (C) of each fibroid. These tissues were immediately frozen and stored at -80°C until analysis for types I, III, and V collagen by classical, stringent collagen extraction techniques. The collagen type I/III ratios were calculated as a classical indicator for tissue remodeling.

#### Collagen extraction and analysis

To extract collagen, 10–30 mg of minced tissue from each sample was incubated overnight at 4°C in 1 ml of freshly prepared 0.1 mg/ml pepsin/0.5 M acetic acid (HAc) solution. The remaining insoluble tissue was removed by centrifugation and subjected to repeated extractions under the same conditions. The collagen yield became negligible in the fourth extract, which was discarded together with the tissue. The first three extracts were combined. Collagen was precipitated by adding sodium chloride (NaCl) to 2 M final concentration, separated by centrifugation, resuspended in 50 mM Tris/0.1 M Na-carbonate/0.5 M NaCl (pH 7.5–8.5), and treated with 0.1 mg/ml pronase for 4–5 h at 4 ^o^C. The pronase treatment was stopped with 0.5 M HAc (final concentration) and collagen was purified by precipitation with 2M NaCl (final concentration). This treatment was utilized to disrupt pepsin-resistant intramolecular cross-links, minimizing the amount of cross-linked α1(I)_2_α2(I) trimers that migrate close to disulfide-bonded α1(III)_3_ trimers on unreduced gels. The purified collagen was fluorescently labeled with amino-reactive Cy5 (GE Health Care) as previously described [29]. Its chain composition was analyzed in triplicate by gel electrophoresis on precast 3–8% Tris-acetate gradient mini-gels (Invitrogen) with and without the reducing agent, Tris (2-carboxyethyl) phosphine (TCEP, Invitrogen). The fraction of each chain was determined from the fluorescence intensity of the corresponding band on the gel. The intensities were calibrated using purified types I, III and V collagen. Globular molecular weight standards are not useful for collagen SDS/PAGE analysis, because collagen chain migration is strongly affected by their high proline content. Only collagen bands were present in these gels and identified by their relative position. Type III collagen chains were identified based on their migration as trimers without TCEP and comigration with α1(I) in the presence of TCEP. To accurately determine the intensities of α1(I) and α2(V) bands that migrate close to each other on the gel, we analyzed depleted and enriched fractions of type V collagen. We purified the type V collagen depleted fraction by selective precipitation of types I and III collagen from 0.5 M HAc solution with 0.7 M NaCl. We purified the type V collagen enriched fraction by subsequent precipitation of the remaining type V collagen with 2 M NaCl. We determined the ratio of α1(I)/α2(I) band intensities by analyzing the type V collagen depleted fraction and the ratio of α1(V)/α2(V) band intensities by analyzing the type V collagen enriched fraction. We then utilized these ratios to recalculate the fractions of α1(I), α2(I), α1(III), α1(V), α2(V), and α3(V) chains in initial samples and thereby determine the fractions of types I, III and V collagen in extracts from different fibroids.

### Statistics

Tissue stiffness was determined as the average of the measurements from 2–3 punches from each sample. Stiffness data measured in Pascal [Pa] is presented in the results as mean ± SD. Stiffness in fibroid samples ranged widely and therefore the variability was also expressed as CV (coefficient of variation calculated as the standard deviation divided by the mean). This statistic describes the percent standard deviation from the mean and allows for the relative comparisons of variability even if means are considerably different from one another. Analysis of variance (One-way ANOVA) followed by Sidak’s multiple comparison test was performed using GraphPad Prism (La Jolla, CA) to compare stiffness among fibroids. Differences were considered significant at *P ≤* .05.

Pearson’s correlation coefficient was calculated for tissue stiffness and collagen content using the formula function in Microsoft Excel 2016.

## Results

### Gross anatomy reveals diverse architectural patterns

**Fig 1:**
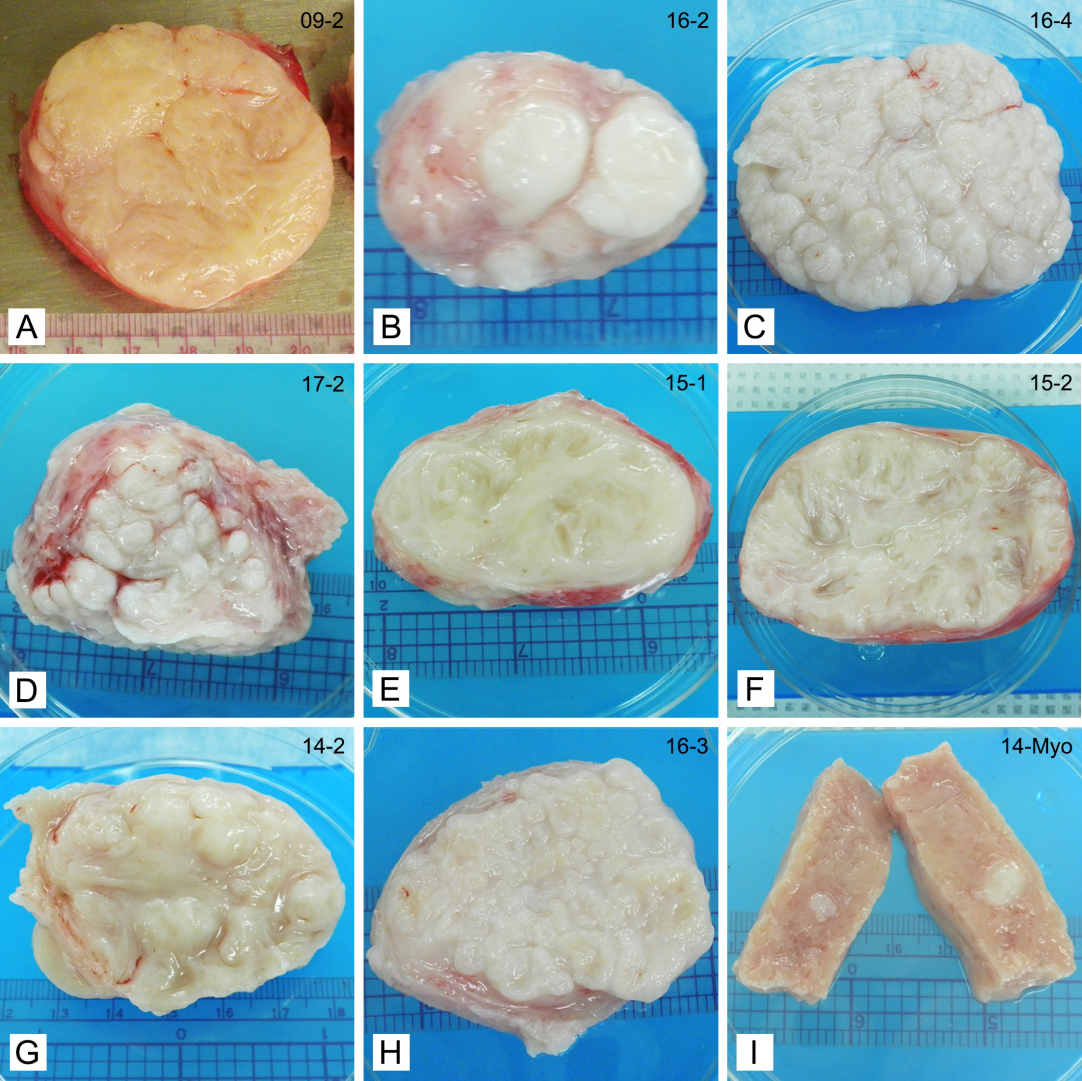
Representative photographs of tissue slices showing differences in gross appearance of fibroids. **A:** Classical irregular whorled pattern; **B, C, D:** Patterns of nodules; **E, F:** Trabecular structures; **G:** Characteristics of multiple patterns. This example shows a trabecular/nodular pattern; **H:** Not categorized. This example shows a tightly gyrated pattern. **I**: Myometrial tissue shown for comparison. Note the seedling fibroid embedded in the tissue (white appearance). Ruler (cm) shown for size.

On the cut surface of the 19 tumor slices studied, we observed a spectrum of tissue architectural patterns. Eight fibroids displayed the classical whorled pattern traditionally described in textbooks (Fig 1A). In nine fibroids, we observed a nodular pattern with small and large nodules. Upon cutting the slices, most nodules immediately protruded above the cut surface. These nodules varied in size from 2 to 14 mm and were stiffer to palpation than surrounding areas (Fig 1B, 1C and 1D). In nine fibroids we observed an interweaving trabecular pattern (Fig 1E and 1F), and six fibroids displayed characteristics of two or more of these patterns (Table 1 and Fig 1G). Two fibroids could not be assigned to one of the three main categories. In one of these fibroids we observed a pattern reminiscent of gyri in brain tissue (Fig 1H). Myometrial tissue is shown for comparison (Fig 1I). This particular sample contained the coincidental finding of a small seedling fibroid that was firm to palpation. In summary, we identified at least three distinct architectural patterns in fibroids and also observed patterns not commonly described. Some fibroids displayed multiple patterns.

**Table 1:** Characteristics of examined fibroid tissue slices.

Fibroid Slice	Diameter [cm]		Appearance		Collagen [%]	Stiffness [Pa]	Stiffness CV [%]
13–1	3.7	Whorled		Nodular	47	3028	33.0
10–2	3.0	Could not be classified			56	3716	34.9
14–2	5.7		Trabecular	Nodular	49	4228	1.6
15–2	8.5		Trabecular		43	5304	18.7
9–2	5.0	Whorled			48	5569	2.4
9–3	11.0	Whorled	Trabecular	Nodular	55	6877	40.5
9–5	7.0	Whorled			65	6994	42.9
16–1	4.5			Nodular	45	7057	22.1
15–1	6.5		Trabecular		53	7276	8.7
17–1	6.0	Whorled	Trabecular		49	7325	13.9
12–1	8.0		Trabecular		71	7348	12.8
16–4	7.5			Nodular	48	8570	20.5
16–2	4.0			Nodular	64	9792	42.0
17–3	4.5		Trabecular		37	10035	29.0
14–1	5.4	Whorled			67	10210	27.4
16–3	5.5	Could not be classified			63	10251	14.1
14–4	8.5	Whorled	Trabecular	Nodular	45	11126	25.9
14–3	6.0	Whorled	Trabecular	Nodular	77	11286	29.9
17–2	3.8			Nodular	49	14180	19.4

### Masson trichrome staining (collagen content)

**Fig 2:**
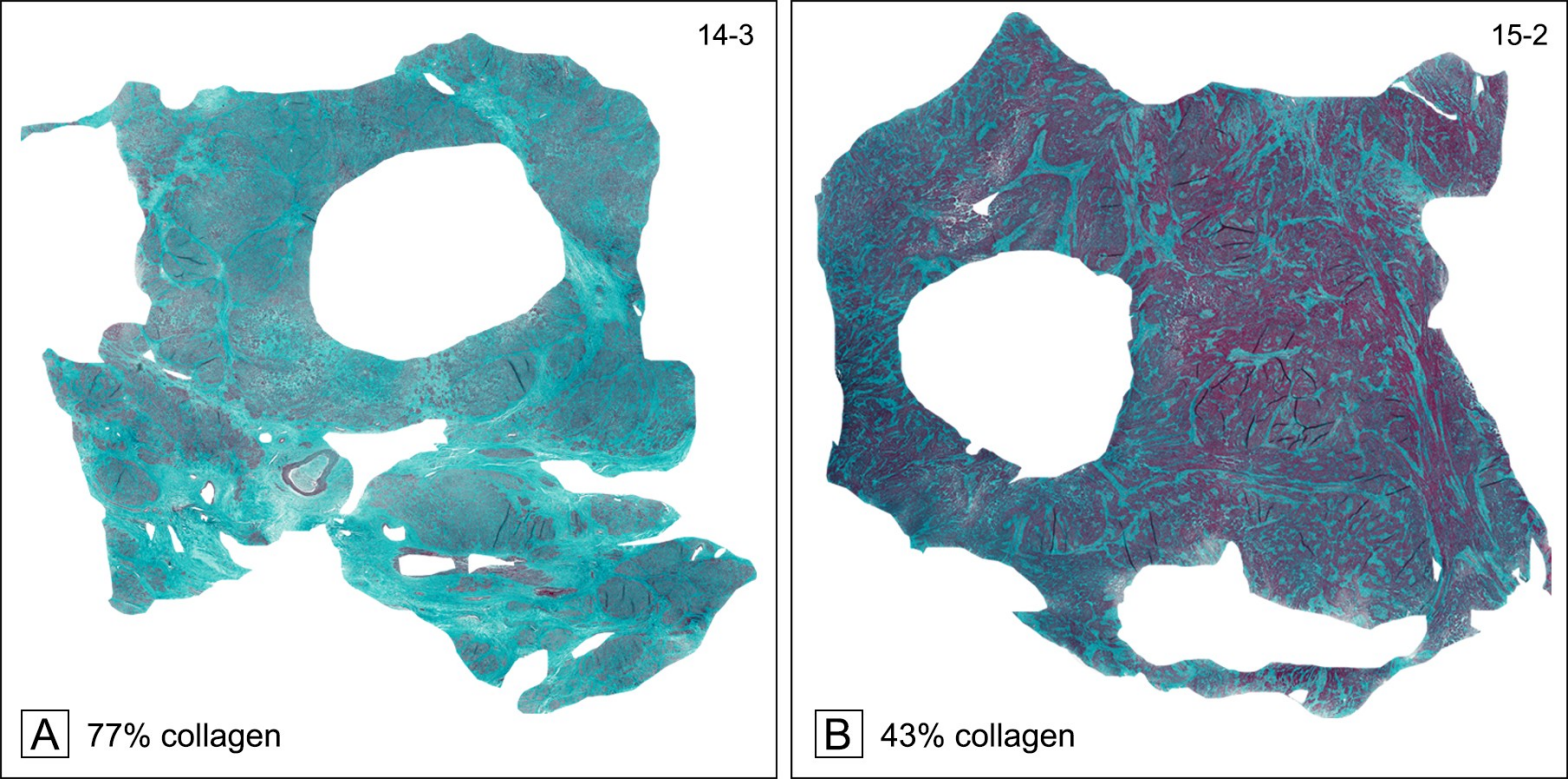
Representative samples of Masson trichrome-stained fibroid tissues (collagen stained blue-green; muscle cells stained red) examined under digital microscopy (20x). Samples (approx. 1x1 cm) from 2 different fibroids were chosen representing a high collagen content (A:14–3) and a relatively low collagen content (B:15–2). The circular holes are due to 5 mm punches taken for rheometry before samples were fixed and stained. Collagen was quantified using pixel counts and is denoted underneath each sample.

We found an abundance of positive Masson trichrome staining in fixed tissues and confirmed that collagen is a large component of uterine fibroids. Tissue samples (approximately 1x1 cm) from each fibroid, had been stained with Masson trichrome and the entire section was captured as a digital microscopic scan (Fig 2). The representative images in Fig 2 were chosen to show examples of high and low collagen content with a similar overall shape of the tissue section for better direct comparison. The circular holes visible in each sample in Fig 2 are due to 5 mm punches taken for rheometry before samples were fixed and stained for collagen. The entire tissue area from each sample was used for analysis and contained on average 3.5 x 10^8^ ± 2.4 x 10^7^ pixels (mean ± SEM). All examined fibroid slices contained at least 37% collagen and collagen staining varied widely (Fig 2 and Table 1). Fibroid size was not correlated with collagen content (correlation coefficient = 0.065).

### Mechanical stiffness highlights fibroid variability profile

**Fig 3:**
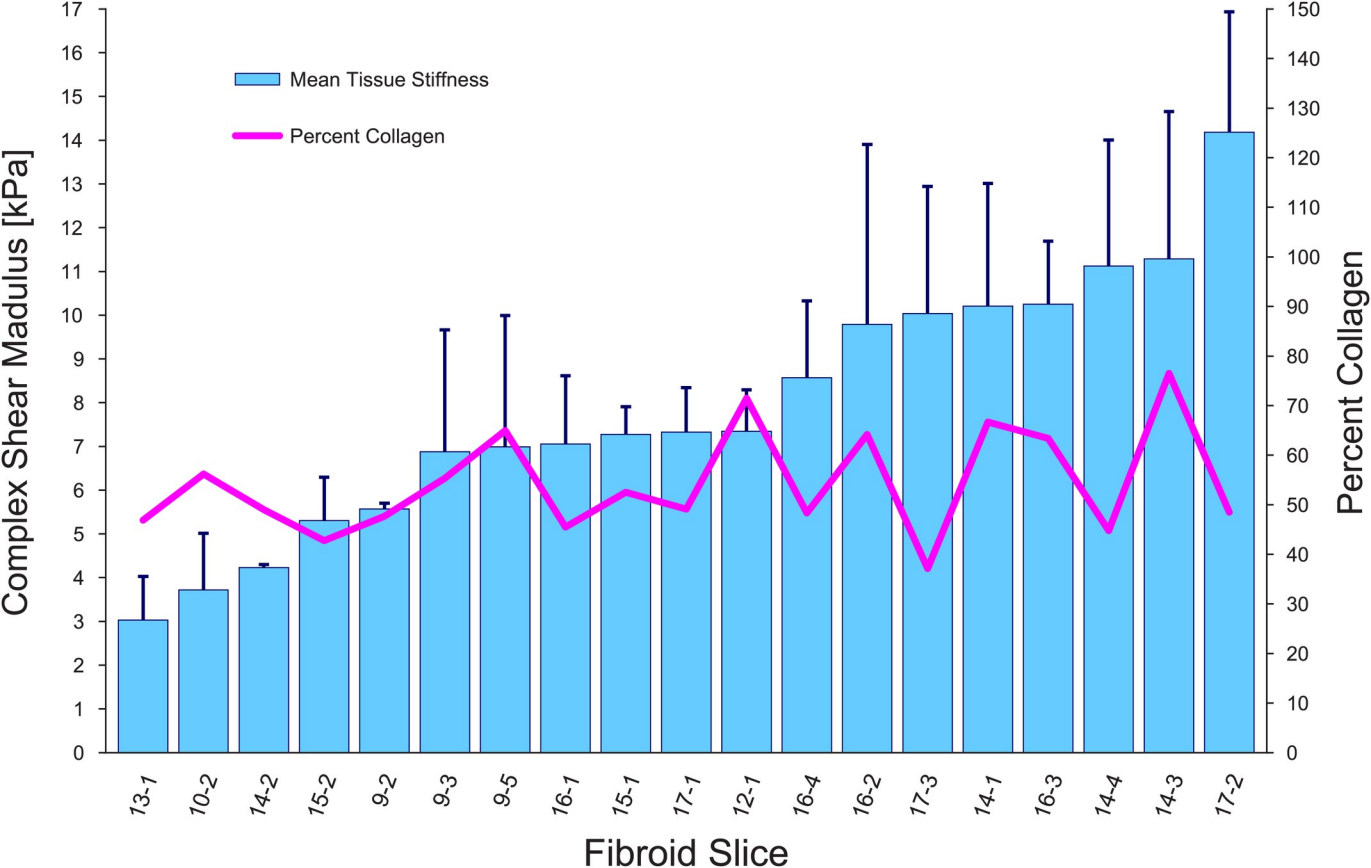
Stiffness and percent collagen in fibroids. Columns represent mean tissue stiffness (complex shear modulus [kPa]) in 19 fibroid slices from 8 different subjects. X-axis labels indicate the subject number followed by the fibroid number. Five subjects contributed more than one fibroid to the study. Error bars indicate within-fibroid variability (SD). The pink line represents percent collagen in each fibroid slice as determined by analysis of Masson trichrome staining. The correlation coefficient of stiffness to percent collagen was 0.22.

A total of 44 samples were measured by rheometry utilizing settings previously used in fibroid tissues [6]. Stiffness among all individual tissue punches (within and between fibroids) varied widely (range = 2027–16130 Pa; mean = 7628 Pa; median = 7216 Pa; SD = 3254 Pa; CV = 42.7%). Data reported in Table 1 lists the sample averages from the 2–3 punches from each fibroid slice. Averages ranged from 3028 to 14180 Pa (Table 1 and Fig 3; CV 36.7%; p<0.001, one-way ANOVA), and revealed among-fibroid variability. Within-fibroid variability is visualized by the error bars (SD) in Fig 3; standard deviations ranged from 70 to 4110 Pa (Fig 3; CV 1.6 to 42.9%, median CV 22.1%). We also observed within-subject variability in the five subjects with more than one fibroid (SD 800 to 3500 Pa; CV 12.2 to 36.4%). For example, the three fibroids from Subject 17 have stiffness values ranging from 7325 to 14180 Pa (Table 1 and Fig 3). Interestingly, fibroid stiffness was neither correlated with the percent collagen content (Fig 3; correlation coefficient = 0.22), nor with fibroid size (correlation coefficient = 0.002).

### Type I, III, and V collagen content in five fibroids

**Fig 4:**
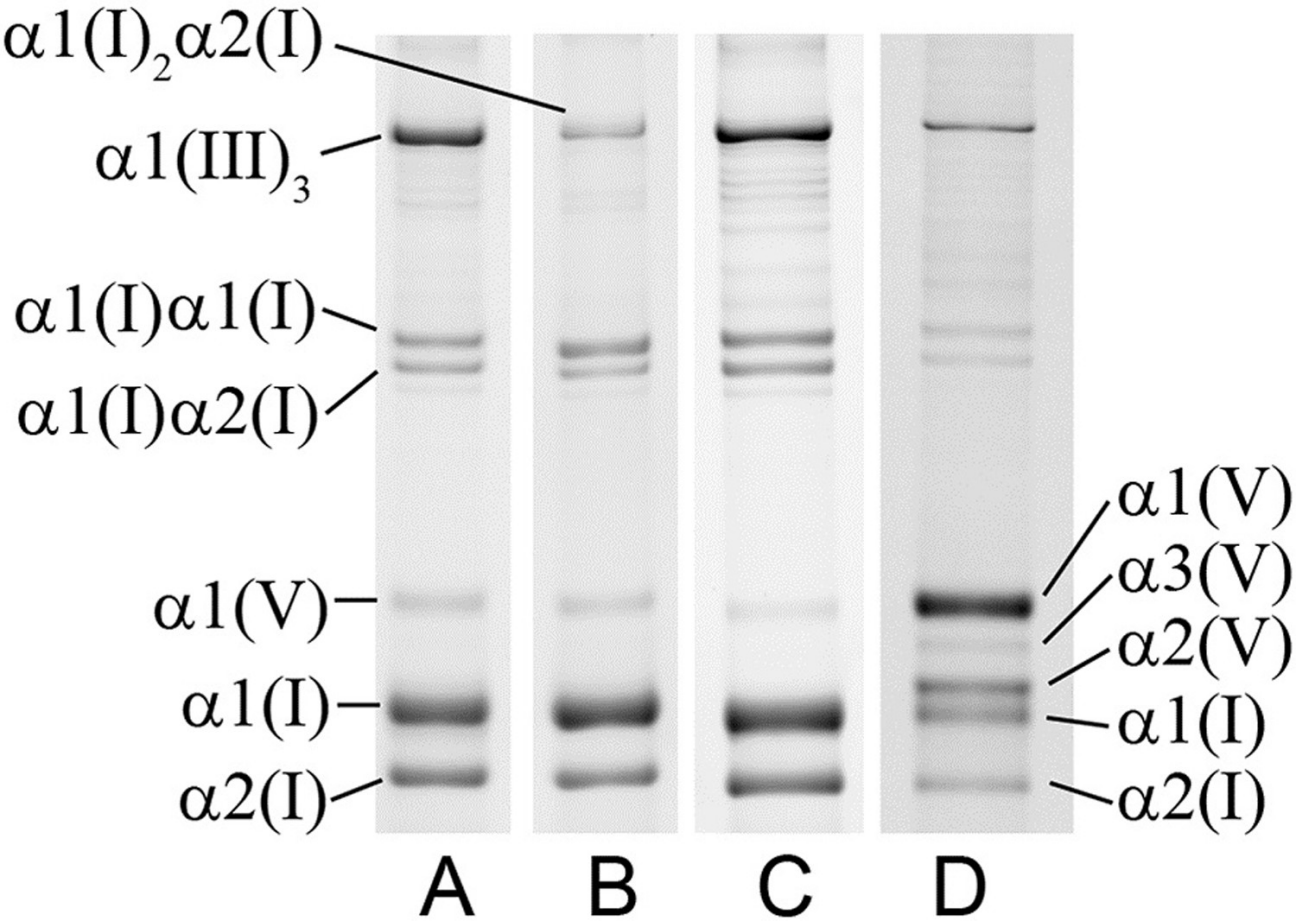
SDS-PAGE analysis of collagen in a representative fibroid sample. **Lane A**: Total collagen extract under non-reducing conditions. **Lane B**: Total collagen extract under reducing conditions (with TCEP). **Lane C**: Collagen extract depleted of type V by selective salt precipitation. **Lane D**: Collagen extract enriched in type V by selective salt precipitation. Sample shown is from 395-E.

The examined fibroid tissues, taken from the center (C) and edge (E) of each of five additional subjects, were studied by classical, stringent collagen extraction techniques. They contained interstitial collagens types I, III, and V of different proportions (Fig 4 and Table 2). While type V collagen was found in all examined fibroid samples, type I and type III collagens were predominant. The proportions of types I, III, and V collagen varied among fibroids samples and ranged from 37–74%, 22–55%, and 2.0–7.4%, respectively. In 4 out of 5 fibroids type I collagen was the major component, but in one fibroid sample (#8), type III was present in higher amounts than type I.

**Table 2:** Proportion of collagen types in fibroids.

Fibroid			Collagen Types [%] (average ± standard deviation)			
	Fibroid Size [cm]	Sample*	Type I	Type III	Type V	Ratio Type I/III
**5**	**4 x 4**	**Center**	**73 ± 16**	**24 ± 6**	**2.0 ± 0.3**	**3.0:1**
		**Edge**	**65 ± 5**	**32 ± 2**	**2.1 ± 0.2**	**2.0:1**
**8**	**8 x 8**	**Center**	**37 ± 1**	**55 ± 1**	**7.4 ± 0.6**	**0.7:1**
		**Edge**	**42 ± 7**	**49 ± 6**	**7.0 ± 1.0**	**0.9:1**
**395**	**5.5 x 4**	**Center**	**63 ± 17**	**32 ± 6**	**4.0 ± 0.9**	**2.0:1**
		**Edge**	**68 ± 13**	**28 ± 4**	**2.7 ± 0.6**	**2.4:1**
**401**	**9.7 x 2.8**	**Center**	**74 ± 13**	**22 ± 4**	**2.8 ± 0.6**	**3.4:1**
		**Edge**	**68 ± 3**	**29 ± 1**	**2.6 ± 0.2**	**2.3:1**
**411**	**12.5 x 10.5**	**Center**	**58 ± 19**	**38 ± 8**	**3.1 ± 0.8**	**1.5:1**
		**Edge**	**72 ± 19**	**26 ± 5**	**2.2 ± 0.5**	**2.8:1**

*Ten samples from five fibroids were studied. Samples were taken from edge and center of each fibroid.

## Discussion

Previously, we and others have reported on the abundant extracellular matrix, especially collagen and glycosaminoglycans content in fibroids and their contribution to mechanical signaling mechanisms and fibroid stiffness [7, 8, 10–13, 15]. In this paper, our observations provide novel evidence that fibroid structural properties and collagen content vary widely. The variations we found in gross appearance of uterine fibroids were striking. In addition, large differences in collagen content and composition as well as stiffness were noted both within and among individual fibroids. Variations in fibroid biology may be associated with different stages of growth and underlying differences in gene expression, protein synthesis, and mechanical signaling and other second messenger production or release. Increased awareness of these differences and intentional consideration of these variations when designing studies and interpreting data will lead to a better understanding of the etiology and pathophysiology of uterine fibroids. The findings reported here lead to the generation of hypotheses ripe for investigations.

Early research involving uterine fibroids has mostly focused on the cellular components of fibroids. Now, the important role of the ECM in fibroid growth has been increasingly accepted [30, 31]. Our study validates that fibroids contain a large percentage of interstitial collagens [9, 10], substantiating that these proteins are an important component of uterine fibroids. Understanding the collagen content, composition, and metabolism in fibroids should greatly improve our overall understanding of uterine fibroid etiology and pathophysiology. Our findings of high variability in collagen content within and among fibroids indicate that collagen metabolism in these benign tumors is active, a fact suggested by ourselves and others [30, 31], and that this metabolism also varies from fibroid to fibroid. Furthermore, we report here for the first time that in several individuals with more than one fibroid stiffness varied among their fibroids, strongly suggesting that in addition to systemic hormonal milieu, local conditions and mechanotransduction may determine fibroid development, growth, and regression.

Cells sense the physical force surrounding them and translate this force into biochemical signals that modulate biological responses. (reviewed in [32]). The mammalian cell responds to physical cues such as stiffness in its environment through a complex system of ECM receptors and transmembrane molecules that interconnect with the cytoskeleton, integrin subunits, and surface glycoproteins (reviewed in [13]). The process of mechanotransduction is dynamic and reciprocal and is as important as traditional biochemical signaling. The ECM stiffness alters signaling within the cell while the cells in turn can modulate the ECM, remodeling the matrix to be either stiff or flexible.

Mechanical forces within collagen-rich fibrotic tissue are known to stimulate cells to secrete more collagen and other components of the ECM. Subsequently, cells develop resistance to programmed cell death (apoptosis) which leads to the persistence of cells and continued secretion of collagen [33]. Mechanical forces consisting of highly cross-linked collagen surrounding individual cells act as localized stimuli for changes in cell biology and behavior, including gene expression. [13, 34, 35] The size of the fibroids in our study ranged from 3 to 11 cm in diamenter and we found significant amounts of collagen in fibroids regardless of size. In uterine fibroids, the degree of hydration and osmotic forces and glycoaminoglycans while not a focus of this paper also play a part in mechanotransduction. [34–36].

Multiple gene expression studies have been carried out with variable results. Some studies suggest that the wide range of expression profiles are due to subtle differences in the characteristics of subjects or laboratory conditions [16]. Fibroids are of clonal origin and certain variations and mutations in specific chromosomes have been found in some fibroids but not in others, revealing genetic heterogeneity among tumors [2, 17]. Whole genome sequencing has reported three genetic triggers of fibroids: FH inactivation, HMGA2 overexpression and COL4A5 and COL4A6 deletion [37]. In addition, two recent studies found MED12 mutations in up to 70% of fibroids examined [18, 19], but a similar study revealed remarkable genomic heterogeneity [20]. It would be very interesting, to perform future well-designed studies of genetic analyses on different nodules within the same fibroid and on different areas within our non-nodular phenotypes to better understand the cellular lineage of these regions with such different macro-appearances. Through focal adhesions and stress fibers leading to the nucleus, alterations in gene expression can be part of the process of mechanotransduction (discussed in [13, 32]) and investigators are beginning to understand the precise mechanisms of how mechanical clues are transduced to the nucleus to influence gene transcription [38]. Variations in fibroid biology may be associated with differences in genetic and non-genetic initiation factors, stages of growth, and, ultimately, gene expression, protein synthesis, and second messenger production or release induced by mechanotransduction. The localized process of mechanotransduction causes individual fibroid cells to change behavior in discrete areas of fibroids. This creates intra-fibroid tissue variability in gene and protein expression, collagen accumulation of different types, and cytokine release. Our findings demonstrate that it is necessary to design more detailed studies investigating and correlating biochemical and mechanical properties within specific areas of fibroids (e.g., nodules and trabecula). It is interesting to note that distinct spatial differences in expression of vascular endothelial growth factor (VEGF) were reported a decade ago. [39]. Our laboratory also found evidence (based on microarray data) that gene expression within the same fibroid can vary depending on location. We had reported differences in the expression of 15 genes between two differing regions analyzed [40], and we speculate that these could be due to differences in the underlying localized pathophysiology as a result of mechanical factors. Increased understanding of differences in gene expression within and among fibroids may assist in the development of targeted therapies.

It has been reported that uterine fibroids grow at different rates within the same woman, and spontaneous regression of these benign tumors can occur. [41] Furthermore, fibroid size does not predict growth rate. [41]. Currently there is no conclusive way to identify if fibroids are growing or shrinking. Attempts have been limited to the description of location or size. Studies designed to determine the exact characteristics of fibroid growth and to determine the growth status of surgically obtained tissue are needed and will advance the field. Future studies of fibroid growth we believe should take mechanotransduction into consideration.

When sliced, considerable variation in gross appearance of fibroids became apparent. Not only did we observe the whorled pattern traditionally described in textbooks, but we also saw distinct nodular, trabecular, and combination patterns. We postulate that underlying differences in biochemistry and thus pathophysiology are responsible for the appearances of the individual samples. For example, one indicator that the tissue was under tension was that nodules immediately protruded from the surface upon cutting. The localized process of mechanotransduction could lead to varied amounts of force exerted on cells in discrete areas of individual fibroids, resulting in structural changes and thus variations in gross appearances.

Interstitial collagen, a major component of the ECM, is one contributor to the stiffness of the matrix. Fibroids have been shown to be stiffer than myometrium in several studies and their results show two to four-fold differences using various measures of mechanical properties [6–9]. However, although all fibroids examined in this study contained large amounts of collagen, stiffness was not correlated with the percentage total collagen (Fig 4). The reason for this remains unclear. Increases in collagen cross-linking contribute to the biomechanical properties of stiffness in fibroid tissue [6, 8], and a recent study has shown that uterine fibroids contain more collagen cross-links than surrounding myometrium. [15]. Higher levels of glycosaminoglycans in uterine fibroids compared to surrounding myometrium also contribute to their stiffness. [8, 11, 13]. We hypothesize that despite similar levels of collagen, fibroid samples may vary in stiffness due to different amounts of cross-linking, glycosaminoglycans, or water content. Further investigation of this hypothesis will reveal the effect that content and characteristics of collagen and other ECM components have on fibroid phenotype and physiology.

Collagen accumulation in tissues is also a hallmark of many localized fibrotic diseases and systematic fibrosis. This collagen accumulation occurs after injury and wound healing or other mechanical stimuli. Masson trichrome does not allow for the determination of the types of collagen present or the amount of cross-linking of the accumulated collagen molecules. The uterine myometrium contains some type IV collagen found in blood vessels, but the most predominant collagens are the interstitial types I, III and V collagen [42]. Uterine fibroids arise from the myometrium and thus these same collagen types are prominent in these tumors. Genes of other collagen types have been reported in microarray studies of uterine fibroids and their adjacent myometrium, [43] but no previous studies have reported biochemical evidence of mature interstitial collagen proteins. Using classical techniques of pepsin digestion, serial precipitation of collagen by NaCl gradient, and separation on SDS gels, the types of interstitial collagens in five fibroids were determined (Table 2). Not only was there a notable variation in proportions of types I, III and V collagen, there was also a variation in the type I/III ratios. In one of the examined fibroids the main component of the tissue was type III (58%) as opposed to type I collagen, which is typically the main collagen component of almost all tissues. In the same fibroid, collagen type V was also elevated. Elevated type III results in decreased collagen type I/III ratios. Such decreased type I/III ratios, as well as elevated type V, are reported in early granulation tissue and restored in late wound healing in scar formation [44–46]. Our findings support the conclusions of other reports suggesting the involvement of the reparative process in the development of uterine fibroids [30, 47–49].

We conclude that there is considerable variation in total collagen content and interstitial collagen types within and among individual fibroids. In other tissues that have been studied, the fibrotic process involves the release of multiple growth factors, cytokines [45], and enzymes such as metalloproteinases. We hypothesize that the myriad changes in these factors in uterine fibroid tissue are also associated with the fibrotic process in uterine fibroids. Future studies should investigate the expression of growth factors and their association with collagen types to provide a more adequate understanding of the complexity of chemical and biomechanical signaling in fibroids.

Fibroid pathobiology and biochemistry is difficult to study as there is no universally accepted animal model for this tumor [50]. Our understanding of these tumors, therefore, will continue to be based on studies utilizing uterine fibroid tissue obtained from women following surgery. Variation among and within uterine fibroids should be documented and accounted for in the design and conduct of research investigating uterine fibroid pathobiology, as well as in translational studies and clinical trials of potential treatments. Heterogeneity has been described at many levels and especially genetic heterogeneity seems to be an obvious grouping factor. However, this requires additional technical analysis. The structural differences we describe here are easily observed upon collection of the fibroids. We suggest that excellent meticulous annotation and greater precision as to the exact location and characteristics of the studied tissue samples will be essential for the evaluation of the data obtained. Our findings indicate that meaningful results could be masked in studies disregarding variations within and among fibroids by using pooled tissue samples.

Our documentation of the heterogeneity among and within fibroids has important ramifications for the design and interpretation of cell culture studies as well. Studies utilizing cell culture or cell lines reflect only the characteristics of the tumor or the part of the tumor from which the culture or cell line was derived and are thus not representative of all fibroid tumors [51] or all regions within the same fibroid. Fibroids usually contain regions with high amounts of ECM /low cellularity and other regions with greater cellularity; fewer cells can be isolated from the former. Therefore, cell cultures derived from heterogeneous fibroid tissue will be enriched in cells from the high cellularity regions of that fibroid and contain fewer cells from the high ECM regions. Experiments performed with this mixed cell population will not adequately represent the characteristics of the cells underrepresented in this mix. Therefore, we hypothesize that many of the cell culture experiments reported in the literature underrepresent the cells from high ECM areas of the fibroid. In future studies, one must keep in mind that different areas of the same fibroid may be in varied physiological stages of development. Therefore, cell populations may be dissimilar due to differences in the biomechanical signaling environments from which they were derived.

In summary, our study revealed heterogeneity among and within uterine fibroids as revealed by differences in total collagen, collagen types, gross appearance, and mechanical variations. Future research must expand and define these differences in more detail with an aim to understand this heterogeneity and possible correlation with other sources of heterogeneity. Our results emphasize the need for careful annotation of tissue sample procurement, precise nomenclature, and consideration of tissue heterogeneity in the design, interpretation, and comparison of future studies. The findings reported here have led to the generation of hypotheses ripe for investigation. Our understanding of fibroid pathophysiology will be enhanced through the investigation of a) growth factors, collagen content, collagen types, and collagen cross-links to understand the complexity of the chemical and biochemical signaling in fibroid development; b) the correlation of biochemical and mechanical properties to more precisely understand mechanical signaling in uterine fibroids; and c) the mechanical forces involved in fibroid development as affected by the various components of the ECM. With a better understanding of commonalities and differences in uterine fibroids and in different regions within fibroids, study designs can be optimized to evaluate different responses to various treatments and develop targeted therapies.

## Supporting information

S1 Dataset Rheometry data from all 44 individual tissue punches. This excel file “S1_Dataset.xlsx” contains the rheometry data (stiffness as measured by complex shear modulus) from all 44 individual punches. These are the underlying data for averages, SDs and CVs presented in the Results Section and in Table 1 and Fig 3.
(XLSX)
